# The Mu story: how a maverick phage moved the field forward

**DOI:** 10.1186/1759-8753-3-21

**Published:** 2012-12-05

**Authors:** Rasika M Harshey

**Affiliations:** 1 Section of Molecular Genetics and Microbiology and Institute of Cellular and Molecular Biology, University of Texas at Austin, Austin, TX, 78712, USA

**Keywords:** Historical significance of Mu, Mu DNA transposition, Shapiro model, Phosphoryl transfer, Transpososome structure, HIV integrase inhibitors

## Abstract

This article traces the pioneering contributions of phage Mu to our current knowledge of how movable elements move/transpose. Mu provided the first molecular evidence of insertion elements in *E. coli*, postulated by McClintock to control gene activity in maize in the pre-DNA era. An early Mu-based model successfully explained all the DNA rearrangements associated with transposition, providing a blueprint for navigating the deluge of accumulating reports on transposable element activity. Amplification of the Mu genome via transposition meant that its transposition frequencies were orders of magnitude greater than any rival, so it was only natural that the first *in vitro* system for transposition was established for Mu. These experiments unraveled the chemistry of the phosphoryl transfer reaction of transposition, and shed light on the nucleoprotein complexes within which they occur. They hastened a similar analysis of other transposons and ushered in the structural era where many transpososomes were crystallized. While it was a lucky break that the mechanism of HIV DNA integration turned out to be similar to that of Mu, it is no accident that current drugs for HIV integrase inhibitors owe their discovery to trailblazing experiments done with Mu. Shining the light on how movable elements restructure genomes, Mu has also given of itself generously to understanding the genome.

## Review

The birth of a journal devoted solely to mobile genetic elements highlights their explosive presence on the genomic scene. With nearly half of our own genomes made up of these elements and with half a century of accumulated knowledge about their workings, it is only a matter of time before answers to questions about their origin, mechanism of movement, habitat preferences, influence on genome structure and gene regulation, as well as their impact on disease, are fully at hand. The more difficult questions are how these elements contribute to evolution at the level of the organism and whether we owe them our very existence [1].

How did we journey from the lone observations of Barbara McClintock in maize, to witnessing the stunning bounty of these elements throughout the biological world? What twists and turns brought us here? No doubt the stories are many [2], but to me, none more exciting than the story of Mu, a bacteriophage that since the beginning has had a seat at the head of the table of this moveable feast. In this Mu-centric perspective, the focus will be squarely on big ideas born from Mu whose ripple effects were transformative.

### Finding Mu

*There is a tide in the affairs of men*,

*Which, taken at the flood, leads on to fortune*;

Omitted, all the voyage of their life

*Is bound in shallows and in miseries*.

*On such a full sea are we now afloat*.

*And we must take the current when it serves*,

*Or lose our ventures*.

William Shakespeare (in *Julius Caesar*)

Mu was accidentally isolated by Larry Taylor during his graduate studies in Edward Adelberg’s lab at UC Berkeley in the late 1950s, while he was attempting a phage P1 transduction into an Hfr strain. During tests for P1 lysogeny in the resulting transductants, he found that they released a phage that was not P1. It so happened that the *E. coli* K12 Hfr strain he was using was the only strain in Adelberg’s stocks that was lysogenic for Mu. Had he not used this strain, Mu might never have been discovered. The discovery of the new phage, however, was not in and of itself particularly noteworthy; it became so when this phage was found to cause mutations.

**Figure 1:**
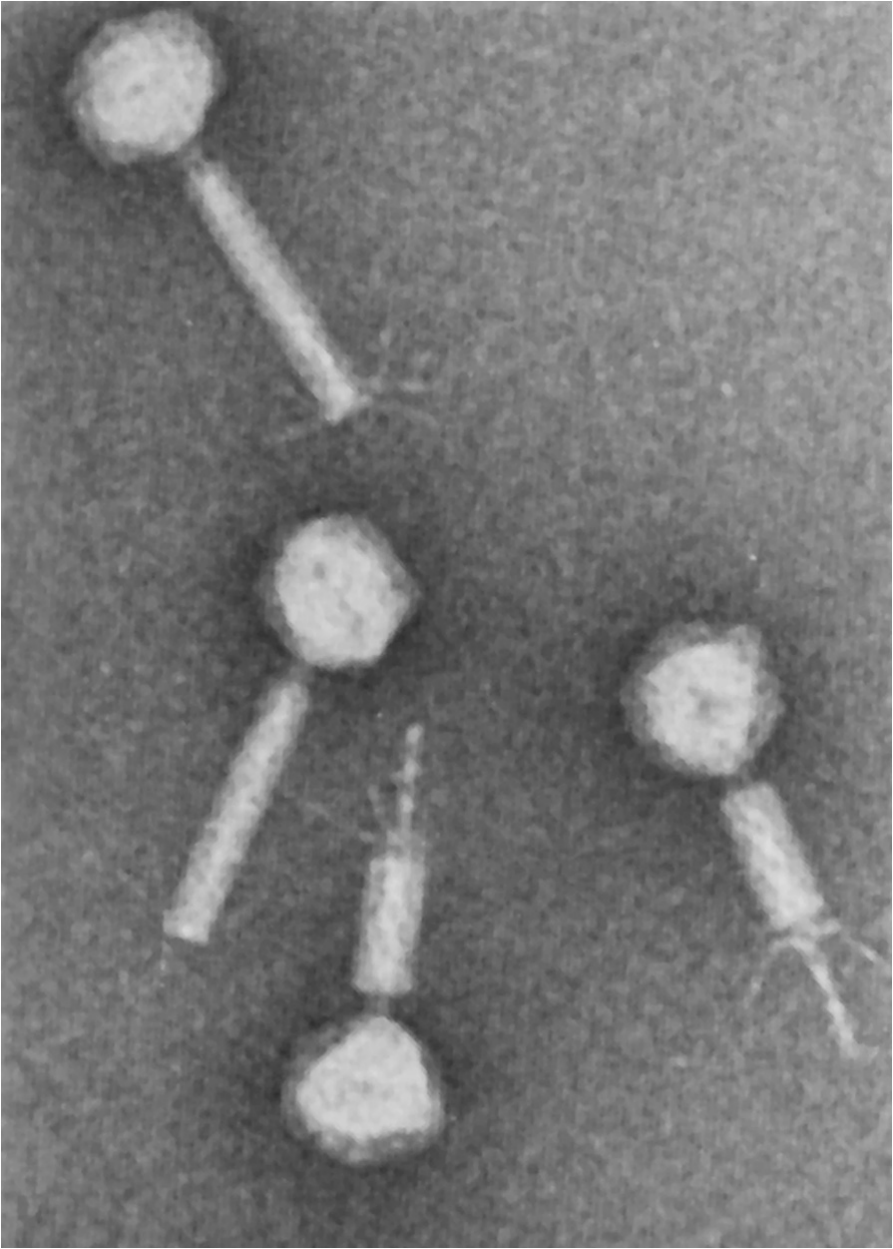
**Electron micrographs of purified Mu phage particles.** This image is reproduced with permission from Martha Howe [4] .

Taylor moved to Milislav Demerec’s lab at the Brookhaven National Laboratory and was setting up some Hfr x F^-^ genetic crosses. The presence of the new phage in the Hfr donor raised a problem of zygotic induction in the recipient. He solved this problem by lysogenizing the recipient strains with the new phage. He spotted the phage on lawns of these strains, which had multiple auxotrophic markers to begin with, picked about 100 colonies from among the survivors of phage infection and carefully checked for all the markers in the strain. He observed that one of the colonies had acquired a new nutritional marker. He disregarded this mutant as a fluke. In the next experiment he again found a colony that had acquired a different nutritional marker. His interest now piqued, Taylor set out to see whether the phage was causing these mutations, and was soon able to show that the nutritional requirements of phage-induced mutants were as varied as those encountered in mutagen-treated bacteria. So he christened the phage ‘Mu’ for mutator. In genetic crosses, he showed that the mutations were inseparable from the prophage, that is, the mutations were caused by the physical presence of the prophage Mu at the affected loci. In the paper reporting this discovery Taylor wrote, “Phage Mu’s dual ability to occupy many chromosomal sites and to suppress the phenotypic expression of genes with which it becomes associated resembles the ‘controlling elements’ of maize more closely than any previously described bacterial episome” [3]. This paper never fails to thrill me as a wonderful example of a completely unprepared discovery by a fully prepared mind (Figure 1).

### Significant Mu discoveries in the 1970s

The precedent for insertion mutations established with Mu influenced interpretation of the curious properties of pleiotropic mutations in the *gal* operon of *E. coli*, whose reversion patterns were inconsistent with those of point mutations, leading eventually to the recognition that they were insertions of Insertion Sequence (IS) elements [5].

Around this time (late 1960s), Ahmad Bukhari, a graduate student in Larry Taylor’s lab at the University of Colorado, began using Mu as a mutagen for experiments related to the biosynthesis of cell wall components in *E. coli*. Fascinated by its properties, Bukhari took Mu with him to Cold Spring Harbor when he established his own laboratory there in 1972. Mu soon became the focal point of his work, which had a far-reaching impact on research into movable elements. At Cold Spring Harbor, Bukhari also came face to face with Barbara McClintock, whose work was vital to his thinking [6]. The first of Bukhari’s many pioneering contributions was the demonstration that Mu could insert randomly at many sites within a single bacterial gene [7]. In a monumental piece of work, he showed that these insertions were extremely stable [8]. On the rare occasions that they reverted, restoration of gene activity was accompanied by excision of the integrated prophage, a formal proof that Mu integration and excision could occur without damaging the target DNA sequences. This also meant that there was a specific mechanism for the recognition of Mu ends, distinguishing them from host DNA. Demonstrations for other transposable elements soon followed and the similar behavior of Mu, IS sequences and other transposons carrying a variety of drug resistances (Tn elements), was the stimulus for a meeting co-organized by Bukhari, Jim Shapiro and Sankar Adhya in 1977, and credited with being pivotal in accelerating research into these elements [9]. This meeting marked the transition point from the idea of a static and unchanging genome to that of a dynamic and mobile one.

The effort now shifted to thinking about how transposable elements move through the genome independent of DNA sequence homology. Clues about mechanism began emerging from accumulating reports of shared chromosomal aberrations - deletions, inversions, replicon fusions - mediated by Mu, IS and Tn elements. Two Mu papers played a critical role in showing that Mu did not excise during transposition, but rather duplicated itself while joining unrelated DNA segments together. The first was a paper by Ljungquist and Bukhari, who induced a Mu prophage to enter into lytic growth and then followed the fate of the prophage-host DNA junctions for the next 30 minutes using the emerging technology of restriction analysis and Southern DNA hybridization [10]. Unlike the control λ prophage, whose original λ-host junctions disappeared soon after induction, consistent with its physical excision, the original Mu-host junctions remained intact late into the lytic cycle, concomitant with the appearance of many new Mu-host junctions. Because Mu was replicating during this time, it was apparent that Mu could move to new sites without leaving its original location. The second seminal paper was by Michel Faelen and Arianne Toussaint, who showed that Mu can join unrelated DNA segments, duplicating itself in the process [11]. The non-excisive, concerted replication-integration mechanism for Mu transposition suggested by these studies served as the archetype for many subsequent transposition models.

### Mu and the Shapiro model for replicative transposition

**Figure 2:**
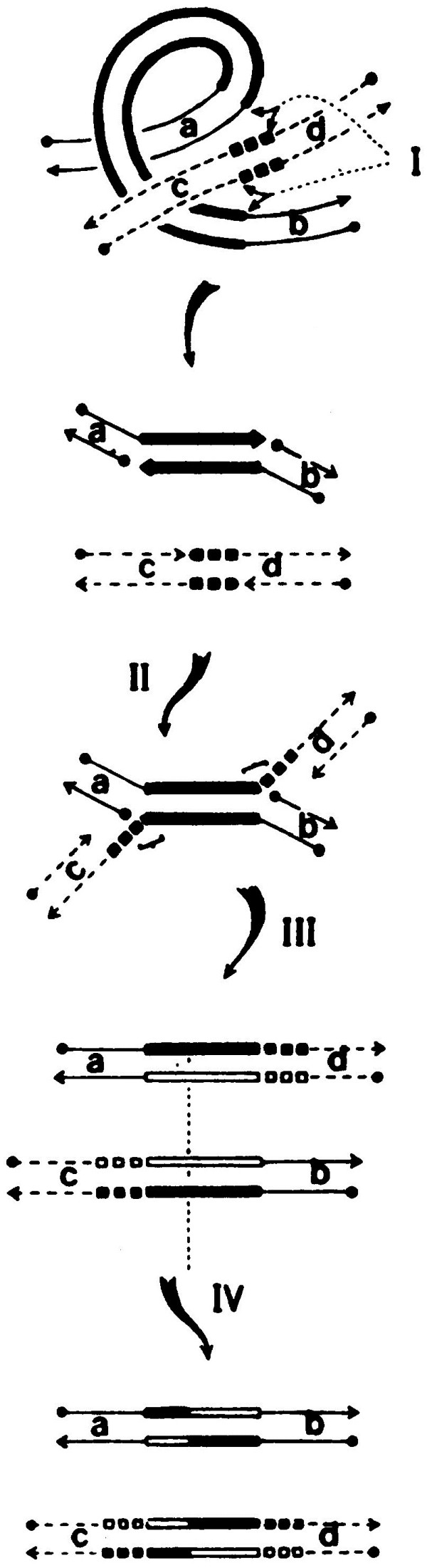
**Shapiro model for transposition of Mu and other elements.** Taken from [12]. The original legend for this figure reads as follows: “Transposition and replication. The top cartoon illustrates how various DNA regions may be brought into close physical proximity for the subsequent cleavage and ligation events. The bottom four drawings show various steps in the transposition process as described in the text. Solid lines indicate donor DNA; dashed lines indicate target DNA; the heavy bars are parental DNA of the transposable element, and open bars are newly synthesized DNA; the small boxes indicate the oligonucleotide target sequence (filled, parental DNA; open, newly synthesized DNA). The arrowheads indicate 3’-hydroxyl ends of DNA chains and the dots indicate 5’-phosphate ends. The letters a, b, c and d in the duplex arms flanking the transposable elements and target oligonucleotide serve to indicate the genetic structure of the various duplex products.” This figure is reproduced with permission from James Shapiro.

In 1979, Jim Shapiro proposed an elegant and parsimonious model for transposition [12] (Figure 2). The model relied heavily on observations made with Mu, particularly the Faelen-Toussaint finding of replicon fusions bordered by Mu copies (also observed for Tn*3* and IS*1*), and the puzzling Ljungquist-Bukhari finding that Mu could replicate its genome without excising from its original location. Shapiro postulated four specific single strand cleavages, two within each end of the donor and two within the target. Those in the target were staggered, as first proposed by Grindley and Sherratt to explain target site duplications associated with IS element transposition [13]. The single strands in the donor were then ligated to those in the target to create a joint molecule, which we now refer to as the ‘Shapiro intermediate’, so resoundingly has the model been verified for Mu. This intermediate created two replication forks at each joint, explaining nicely how Mu transposition and replication could be coupled, and how the Mu replica could simply peel off the original site without Mu excision. The model was strengthened by being able to offer a simple explanation for inversions and deletions of target markers that were always found linked to a copy of the transposon: alternate polarities of the strand joining event would lead to these alternate rearrangements. The model also suggested why transposable elements would likely not have an autonomous existence - a striking feature of Mu virions where each particle has a Mu genome buried within host DNA. The model postulated all reactions to be limited to local regions, with no need for base pairing at the site of insertion. This would be the first example of non-homologous, illegitimate, yet somewhat site-specific recombination. Now, 30 years later, a survey of transposons and transposition mechanisms shows a multitude of different ways of movement, which do not always go through the Shapiro intermediate. But the reaction always initiates with donor cleavage, whether single or double stranded, and always ends in these strands joining to the target, with or without intervening steps of hairpin formation or replication (see [14]).

### The biochemical era

**Figure 3:**
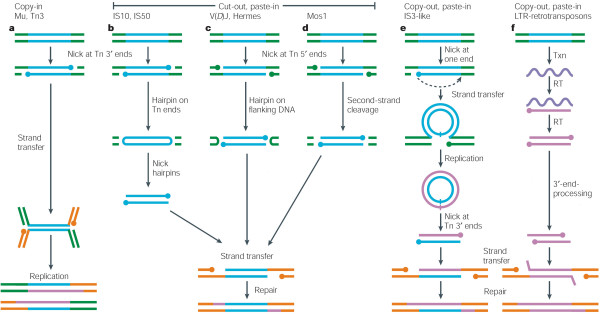
**A multitude of transposition mechanisms.** For details, see [14]. This figure is reproduced with permission from the Nature Publishing Group.

George Chaconas and I were post-docs in Ahmad Bukhari’s lab in the early 1980s, where we constructed mini-Mu plasmids and showed that their transposition behavior *in vivo* was true to that of their Mu parent [15]. We intended to use these convenient substrates to set up *in vitro* transposition reactions in our own labs, when we were blindsided by Kiyoshi Mizuuchi, who had been studying the biochemistry of phage *λ* integrative recombination, and who published the first successful protocol for observing transposition *in vitro* using mini-Mu plasmids [16]. A decade of brilliant experiments from the Mizuuchi group, using either mini-Mu plasmids or Mu end oligonucleotides as donor substrates, laid the foundation for our current knowledge of transposition mechanisms. They revealed the reaction chemistry to be transposase-mediated hydrolysis of a specific phosphodiester bond between each Mu end and its flanking DNA, exposing 3’-OH ends, which then attack target DNA at staggered positions 5 bp apart. Both these steps involve metal-ion mediated activation of nucleophiles - water in the first step and the free 3’-OHs of DNA in the second step – and are direct phosphoryl transfer reactions that do not involve covalent protein-DNA intermediates [17]. The Mu experiments were torchbearers for establishing *in vitro* reactions for other transposable elements (Tn*10*, Tn*7*, Tn*5*, P elements, retroviral elements, VDJ recombination), and it became clear that while details varied, they all shared similar phosphoryl transfer chemistry (see [2]) (Figure 3).

Three important papers contributed to understanding the Mu reaction in the context of a higher-order nucleoprotein assembly. The first was the elucidation of the domain organization of the transposase MuA protein [18], which helped in assigning specific functions to each domain, ultimately paving the way for use of these domains in structural studies. The other two papers came from George Chaconas’s lab, which trapped ‘transpososomes’ in the act of cleavage or strand transfer [19]. A hallmark of the transpososomes is their extraordinary stability, with a catalytic core comprised of a tetramer [20]. These studies facilitated separation of uncleaved, cleaved and strand transfer complexes on agarose gels, greatly expediting the analysis of various *cis* and *trans* factor requirements for each step of the reaction. The complexes get progressively more stable as the reaction proceeds, a feature apparently designed to progressively rearrange the reacting components and prevent chemical reversal of the reaction. Similar multi-subunit complexes and stabilities have been observed for many transpososomes (see [2]).

Another trio of Mu papers established that only two subunits in the multi-subunit MuA transpososome were responsible for catalysis, and that these subunits act in *trans*, ensuring that the reaction was not initiated until both ends were paired and all the players were in place [21-23]. These studies were aided by the use of oligonucleotide substrates that could be preloaded with desired catalytic mutants, or isolation of altered specificity variants of MuA that could be directed to specific binding sites. The *trans* feature of transposase-mediated catalysis is also proving to be widespread among mobile elements [24-27].

### Mu, HIV and the structural era

HIV arrived on the scene in the early 1980s, at a time when avian and murine retroviruses were being actively studied. The resemblance of Long Terminal Repeat (LTR) sequences at the ends of retroviral DNA to those at the ends of transposable elements, and their ability to integrate and cause duplications at the site of insertion, had immediately suggested that these were similar to insertion elements (see 1981 Cold Spring Harbor symposium volumes). Several labs set up *in vitro* integration reactions and identified the integrase (IN) to be responsible. A comparison of a large number of retroviral INs, prokaryotic and eukaryotic transposases as well as the RAG recombinase involved in immunoglobulin VDJ rearrangements, led to identification of conserved D,D(35)E motifs that were shown to be important for integration; these residues coordinate the metal ions required for nucleophile activation [28]. Although MuA did not readily show up in this analysis, it was later also shown to belong to the DDE family of transposases.

**Figure 4:**
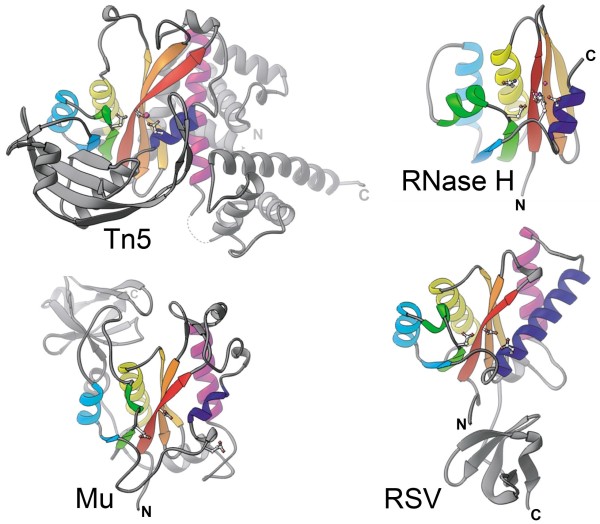
**A conserved transposase catalytic core homologous to the RNase H fold.** Single subunits or fragments of the four indicated family members are shown with conserved secondary structure elements colored similarly. For details see [33]. This figure is reproduced with permission from the Nature Publishing Group.

**Figure 5:**
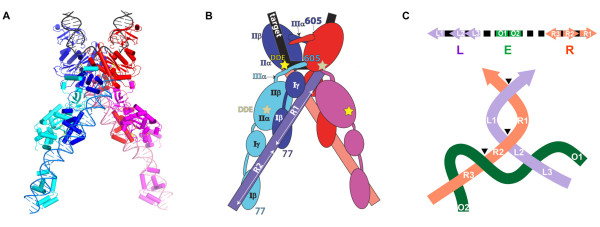
**Crystal structure of the Mu transpososome and Mu DNA topology.** The figures in (**A**) and (**B**) are reproduced with permission from Phoebe Rice [35]. **(A)** Structure of the Mu transpososome engaged with cleaved Mu ends joined to target DNA. **(B)** Schematic of the structure in A, illustrating positions of the various MuA domains and DNA segments. Full length MuA protein is 630 residues long. The polypeptide in the crystal structure includes residues 77 to 605; it is missing the regulatory N (Iα)- and C (IIIβ)-terminal domains that interact with the enhancer, and with proteins MuB and ClpX, respectively. Catalytic sites are marked as tan/yellow stars in B. The donor DNA in the crystallized complex consists of two equivalent right ends, each with two MuA binding sites. It shows one right-handed DNA crossing. (**C**) On native Mu DNA, the left (L) and right (R) ends have three MuA binding sites each, and are non-equivalent with regard to their spacing and orientation. A third enhancer (E) segment is essential for assembly of a functional transpososome. The enhancer is positioned closer to the L end on the Mu genome. In a transpososome assembled on this native configuration of the L, E, R segments, six MuA subunits bound via their Iβγ domains to the L (L1-L3) and R (R1-R3) ends, make bridging interactions with the enhancer (E: O1-O2) via their Iα domains to trap five supercoils - two L-R, two R-E and one L-E crossings - as indicated (see [37]). R-E interactions initiate assembly and are essential [38]. When the transpososome is treated with high salt or heparin, a stable tetrameric core remains, which still retains two L-R and one R-E DNA crossing (black arrow heads) [39]. Of the two L-R crossings, the one at the top of the diagram, is likely the one seen in the crystal structure (A, B). Placement of the second L-R crossing is arbitrary, but note that this crossing as well as the proximal R3-E crossing is maintained by the tetramer [39]. See [38] for details.

The similarities between the phosphoryl transfer steps of Mu and HIV integration are remarkable, both occurring by a one-step mechanism without involvement of a covalent protein-DNA intermediate [29]. Both Mu and HIV have a conserved CA dinucleotide at each end at which cleavage occurs, and both transposase/integrase proteins cut the target in a 5 bp stagger. Both MuA and retroviral integrases are organized in three independently folding domains, with the central domain containing the catalytic DDE residues. The catalytic core domains of both MuA and retroviral integrases were the first to be crystallized and showed a striking similarity in their active sites and overall RNase H structural fold [30-33] (Figure 4). Despite the early success with crystallizing catalytic domains of these proteins, a structure of their transpososomes eluded crystallization. The first such structure obtained was that for Tn*5*[26], and a decade later those for the IS*200*/IS*605* family and Drosophila Mos*1* elements [24,25]. All three structures are dimers. They show a *trans* arrangement of catalytic subunits, as first functionally demonstrated for MuA. The highly interwoven protein-DNA and protein-protein contacts explain the high stability of transpososomes. Structures of intasomes captured at various stages of the integration of the retrovirus PFV (prototype foamy virus), also show a *trans* arrangement of catalytic subunits, with target DNA accommodated in a severely bent conformation in the strand transfer complex [27,34]. Finally, the long-awaited transpososome structure of Mu, the mobile element that started it all, is here [35]. The structure shows the expected *trans* arrangement of the catalytic subunits, with target DNA also bent as in the PFV intasome (Figure 5A,B). Target bending is another feature common to transpososomes [36], and is proposed to contribute to the irreversibility of the reaction by having the strained DNA spring away from the active sites after strand transfer [35].

Modeled after the Mu *in vitro* system, convenient HIV integration assays were developed that could be carried out in the wells of micro-titer plates for screening large numbers of potential inhibitors of HIV DNA integration [40]. Over the years, a plethora of HIV integrase inhibitors have been discovered, but raltegravir is the first drug developed by Merck targeted to the integrase to be approved by the FDA (Federal Drug Administration) [41]. This drug, which inhibits the strand transfer step, was shown in the intasome structure of the related retrovirus PFV to intercalate between the terminal ends, displacing the reactive 3' viral DNA end from the active site, and chelating a metal ion [27]. The PFV structure will be invaluable for the development of next-generation integrase inhibitors.

### Where Mu gets its mojo

Since Mu amplification is dependent on transposition, it has evolved enhancing functions that are peculiar to Mu and Mu-related phages [42]. These include a *cis*-acting transposition enhancer, an accessary transposition-enhancing protein MuB, and a centrally located strong gyrase binding site SGS.

The *cis*-acting enhancer is an essential component of the transposition reaction. It acts as a scaffold during transpososome assembly [43], directing an ordered interaction with the two Mu ends to generate a highly intertwined 3-site synapse which traps 5 DNA supercoils [38,44-46] (Figure 5C). Many regulatory roles for the enhancer have been deduced (see [47]), including serving as a topological filter to ensure the most stable configuration of the transpososome, while guarding against pairing incorrect Mu ends in a cell where multiple copies of Mu are accumulating [48]. The enhancer requirement can be bypassed *in vitro* by use of the solvent dimethyl sulfoxide or high protein and DNA concentrations (see [42]). The recently solved structure of the Mu transpososome does not include the enhancer (Figure 5A, B).

MuB is best known as a protein that enables efficient target selection. It is, however, also a protein with multiple functions, some of them paradoxical. MuB interacts with the C-terminal IIIβ domain of MuA to not only allosterically activate the catalytic potential of MuA and deliver the target to the transpososome, but to also promote target immunity, which prevents some segments of DNA from receiving Mu transpositions (see [42]). Two kinds of target immunity have been uncovered: *cis*-immunity prevents regions in the immediate vicinity of Mu ends from being used as targets, and depends on removal of MuB from these regions [49-51]; Mu genome-immunity prevents Mu from transposing into itself, and appears to operate by a different mechanism where MuB is not only not removed but rather binds strongly [52]. Only one other transposon, Tn*7*, encodes additional target selection proteins [53], while two – Tn*7* and Tn*3* family – display target immunity that resembles Mu *cis*-immunity [54-56].

The SGS site was discovered by Martin Pato and his colleagues, who showed that its central location on the Mu genome was critical for efficient Mu replication [57]. It is the strongest gyrase binding site known, initiating a striking SGS-dependent increase in processivity of the gyrase reaction [58,59]. Pato has proposed that the SGS initiates plectonemic supercoiling at the center that propagates to the Mu termini, enabling efficient synapsis of the ends located approximately 37 kb apart on the Mu genome [60,61].

### Mu replication, non-replicative transposition, and repair of transposition events

Two replication forks are created at each Mu end after strand transfer into target DNA (see Figure 2, II). Transition of the oligomeric MuA tightly bound to the ends, to a replication-ready configuration, has been mainly dissected in Hiroshi Nakai and Tania Baker’s laboratories [62] (Nakai, like Bukhari, was also a graduate student in Larry Taylor’s lab, where he first became interested in Mu replication [63]). In a highly choreographed series of steps, the molecular chaperone ClpX interacts with the C-terminal IIIβ domain of MuA to unfold one of two catalytic subunits, weakening the overall interaction of the transpososome with DNA and allowing exchange with protein factors that ultimately load the restart primosome at the Mu ends for replication [64-69].

It is a quirk of history that the first transposition mechanism analyzed in-depth was the replicative mechanism of Mu. The study of a vast majority of transposable elements that followed showed that they excise and reinsert without replication, like those described by McClintock in maize (Figure 3; see [2]). Not to be outdone on any front, Mu also transposes by a variation of the non-replicative mechanism, but this mechanism is confined to the integration of infecting Mu DNA, that is, Mu injected into the host from a phage particle (see [42]). The initial single strand cleavages at Mu ends and joining to target DNA are the same as in the Shapiro model; however, rather than replication, the transposition intermediate is resolved by removal of 5’ flaps attached to Mu ends, followed by repair of the integrant [70]. This pathway is not yet reproduced *in vitro*, but *in vivo* results show that the flap removal activity is contributed by MuA itself [71,72]. Since every integration event from an infecting Mu virion is non-replicative, this system is poised to reveal cellular mechanisms for repair of such events, of which we know virtually nothing. Recent experiments have already overthrown the long-held assumption that the short gaps in the target left in the wake of transposition are repaired by gap-filling polymerases; for Mu they are repaired by the primary machinery for double-strand break repair in *E. coli*[73]. Given that double-strand break repair pathways have been implicated in repair of the retroviral and LINE retro-element insertions, Mu may once again show us the way.

### Through the Mu looking glass

No discussion of Mu can be complete without citing the pioneering contributions of Malcolm Casadaban, who generated a variety of Mu derivatives as probes for genome transcription and translation, as cloning vehicles, as mobile sources of transcriptional promoters, and as movable primers for DNA sequencing ([74,75]; the two citations included here are book-ends for over a dozen papers from the Casadaban lab). These Mu manipulations were the prototype for a myriad different ways in which transposons have been harnessed as tools for genetic engineering, cleverly using them to deconstruct the genomes they reconstructed [2,76-78].

## Conclusion

Mu was the catalyst that liberated our thinking about transposition, its mechanisms and significance. Ever since Mu, our view of the changing genome has grown enormously, with a superabundance of insertion elements and conjugative transposons, retroviruses and retro-transposons, LINE and SINE elements, homing and retro-homing introns, telomeres and immune system rearrangements. A handful of mechanisms are used over and over in different combinations, generating a great deal of diversity [2,14,79]. What new discoveries can we look forward to in the coming years?

On such a full sea are we now afloat

*And we must take the current when it serves*.

## Abbreviations

F: Fertility factor/episome; FDA: Federal drug administration; Hfr: High frequency recombination; HIV: Human immunodeficiency virus; IN: Integrase; IS: Insertion sequence; LINE: Long interspersed nuclear element; LTR: Long terminal repeat; Mos: Mosaic; P Element: Transposon present specifically in Drosophila; PFV: Prototype foamy virus; RAG: Recombination activating gene; SGS: Strong gyrase site; SINE: Short interspersed nuclear element; TE: Transposable element; Tn: Transposon; VDJ: Variable, diverse, and joining immunoglobulin gene segments.

## Competing interests

The author declares that she has no competing interests.

## Author information

The author is currently a Professor in the Section of Molecular Genetics and Microbiology at the University of Texas at Austin.
